# Lack of association between the HLA region RS7775055 polymorphism and JIA in patients from russia

**DOI:** 10.1186/1546-0096-12-S1-P197

**Published:** 2014-09-17

**Authors:** Victor Malievsky, Tatyana Victorova, Ksenia Danilko, Liliia S Nazarova

**Affiliations:** 1 Department of Hospital Pediatrics, Bashkir State Medical University, Ufa, Russian Federation; 2 Biology Department, Laboratory of Human Physiological Genetics, Bashkir State Medical University, Institute of Biochemistry and Genetics, Ufa, Russian Federation; 3 Biology Department, Central Scientific Research Laboratory, Ufa, Russian Federation; 4 Biology Department, Bashkir State Medical University, Ufa, Russian Federation

## Introduction

Juvenile idiopathic arthritis (JIA) refers to a group of conditions involving joint inflammation that first appears before the age of 16. Juvenile idiopathic arthritis is thought to arise from a combination of genetic and environmental factors. Numerous associations between HLA polymorphisms and JIA subtypes have been reported in multiple populations. The HLA region SNP rs7775055 is a part of the HLA-DRB1*0801–HLA-DQA1*0401–HLA-DQB1*0402 haplotype, which has been shown to increase risk of JIA in UK and non-Hispanic Caucasians US patients. The recent study of Hinks and co-authors also showed the strong evidence of association between rs7775055 and JIA (odds ratio (OR) = 6.01; *p*=3.14×10^−174^) in patients from across the US, UK and Germany.

## Objectives

The goal of the study was to test the hypothesis that the HLA region SNP rs7775055 could underlie susceptibility to JIA or its subtypes in patients from Russia.

## Methods

The HLA region SNP rs7775055 was studied in 204 children with JIA and 207 healthy individuals, citizens of the Bashkortostan, Russia using real-time PCR. Statistical analysis was performed using Statistica v.6.0 and SNPStats programs.

## Results

The genotypes distribution was in Hardy-Weinberg equilibrium in both groups. The HLA region SNP rs7775055 did not show an association either with JIA or any of the JIA subtypes in our cohorts.We revealed a trend tendency towards an increase of CC genotype under a recessive model in persistent oligoarthritis cohort, but it was not statistically significant (*p*=0,068, OR=6.88 95%CI 0,71-67,02).

## Conclusion

In summary, in this study we found no evidence of association between the HLA region SNP rs7775055 and JIA or its subtypes in patients from Russia.

## Disclosure of interest

None declared.

